# POSS and SSQ Materials in Dental Applications: Recent Advances and Future Outlooks

**DOI:** 10.3390/ijms24054493

**Published:** 2023-02-24

**Authors:** Jan Ozimek, Izabela Łukaszewska, Krzysztof Pielichowski

**Affiliations:** Department of Chemistry and Technology of Polymers, Cracow University of Technology, Warszawska 24, 31-155 Cracow, Poland

**Keywords:** polyhedral oligomeric silsesquioxanes, POSS, silsesquioxanes, SSQ, dental composites, nanocomposites

## Abstract

Recently, silsesquioxanes (SSQ) and polyhedral oligomeric silsesquioxanes (POSS) have gained much interest in the area of biomaterials, mainly due to their intrinsic properties such as biocompatibility, complete non-toxicity, the ability to self-assemble and to form a porous structure, facilitating cell proliferation, creating a superhydrophobic surface, osteoinductivity, and ability to bind hydroxyapatite. All the above has resulted in new developments in medicine. However, the application of POSS-containing materials in dentistry is still at initial stage and deserves a systematic description to ensure future development. Significant problems, such as reduction of polymerization shrinkage, water absorption, hydrolysis rate, poor adhesion and strength, unsatisfactory biocompatibility, and corrosion resistance of dental alloys, can be addressed by the design of multifunctional POSS-containing materials. Because of the presence of silsesquioxanes, it is possible to obtain smart materials that allow the stimulation of phosphates deposition and repairing of micro-cracks in dental fillings. Hybrid composites result in materials exhibiting shape memory, as well as antibacterial, self-cleaning, and self-healing properties. Moreover, introducing POSS into polymer matrix allows for materials for bone reconstruction, and wound healing. This review covers the recent developments in the field of POSS application in dental materials and gives the future perspectives within a promising field of biomedical material science and chemical engineering.

## 1. Introduction

For over twenty years, dentistry has been using composite polymeric materials, mainly methacrylate resins, that have outperformed amalgams, ceramics, and metals [1]. The primary use of polymeric materials was initially the construction of prosthetic appliances, such as denture bases [2]. However, nowadays, materials for dentistry consist mainly of polymeric matrixes with possible applications as dental adhesives (acrylic acid copolymers), dental pulp and denting regeneration (poly(lactic acid)) [3], drug delivery systems (polysaccharides, polyketals, polyethylene glycol) [4,5], friction-reducing coatings for biomaterials (zwitterionic polymers) [6], implants (chitosan-poly(methyl methacrylate) blends) [7], soft lining materials (silicone rubbers and soft acrylics), and prosthetics (polyetheretherketone) [2].

To meet the requirements for hydrolytic stability, bioactive/biomimetic and antibacterial capabilities, abrasion resistance, long-term mechanical strength, and other properties, modification of polymeric matrices is usually needed, e.g., by introducing fillers to form composites [1]. Thus, composite resins became the most extensively utilized materials in dental applications. Fillers used in dental composites comprise mainly silica-based materials [8,9]. Engineered silica nanomaterials, such as silica nanoparticles, silica-based nanoclusters [8], silica nanoplatelets, and polyhedral oligomeric silsesquioxanes exhibit biocompatibility and proper physical and mechanical properties, which render them suitable for dental applications [9,10]. 

**Figure 1:**
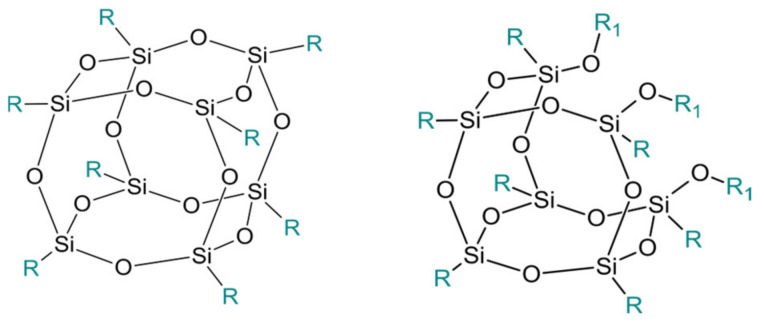
The general structure of closed-cage POSS (**left**) and partially opened-cage POSS (**right**). *R* represents organic substituents functionalizing the POSS cage, while *R_1_* represents organic or silica-organic substituents.

Polyhedral oligomeric silsesquioxanes (POSS) are organosilicon hybrid materials with a three-dimensional, chemically substituted, and thermally stable inorganic silicon-oxygen core (cage) to which organic substituents are covalently bonded [11]. The substituent can be an inert group, e.g., H, alkyl, aryl, and other hydrocarbons, or potentially reactive group, e.g., hydroxyl, amino, and other reactive groups [12]. The general structures of closed-cage and partially opened-cage POSS are shown in Figure 1.

Polyhedral oligomeric silsesquioxanes exhibit unique properties, such as biocompatibility, non-toxicity, stability, tastelessness, well-defined structure, and hydrolytic degradation to neutral orthosilicic acid [12,13,14]. It is noteworthy that orthosilicic acid, in similarity to silica nanoplatelets, stimulates osteoblast differentiation [9,15]. Moreover, POSS exhibits long-term stability and low cytotoxicity in biological environments [16]. 

The most significant advantage of POSS is the ability to tailor their properties by selecting proper substituents [17,18]. Changing the size or type of attached molecules allows for obtaining POSS derivatives characterized by different properties, making POSS a useful nano-additive with a broad range of possible applications [19]. The type of organic substituents attached to the POSS cage core determines the properties of the silsesquioxanes themselves and the properties of the materials in which they will be incorporated [20]. Inert groups can improve the solubility of POSS in polymer systems to make it compatible and miscible with given polymer matrix, while reactive groups act as linkers for grafting or copolymerizing POSS into a polymer structure [12]. 

Introducing POSS particles into a polymer matrix may enhance the material’s toughness, strength, thermal stability, ultraviolet steadiness, abrasion resistance, hardness, and biocompatibility (improved cell attachment and cell proliferation) [21,22]. In addition, silsesquioxane-based composites show reduced flammability and enhanced oxidation resistance compared to unmodified matrices [16,23]. Incorporation of POSS into a polymeric matrix may facilitate in vivo hydroxyapatite formation and render bioactivity [16]. 

**Figure 2:**
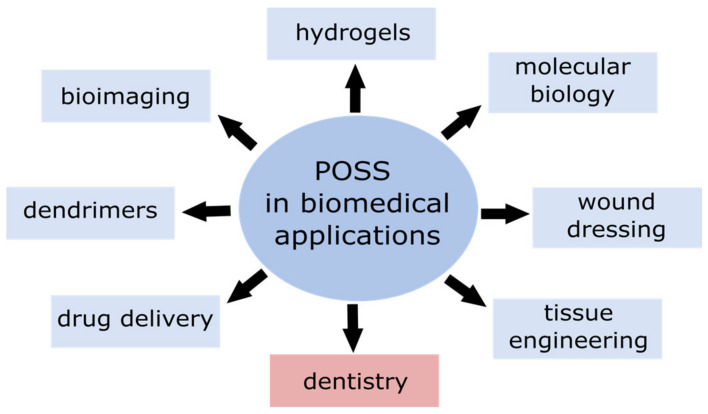
Possible applications of POSS in biomedical fields.

All the above explains considerable interest that POSS have gained in terms of application in biomedical fields (Figure 2), e.g., POSS particles and POSS-based composites are promising for their use in drug delivery, bioimaging, photodynamic therapy, tissue engineering, tissue regeneration, and dentistry [24,25,26,27]. 

Much of the attention devoted to POSS and POSS-based composites in biomedicine concerns the research in the field of dentistry. In this review we present and discuss the most recent advances in the area of POSS and POSS-based polymer composites as materials for dental applications. This work provides also an outlook for the future of POSS/polymer composites applications in dentistry and dental surgery.

## 2. POSS in Dental Materials

Methacryl-functionalized POSS (MA-POSS) has been widely studied as an **anti-shrinkage** additive for dental materials since Culberston et al. introduced MA-POSS to dental composites in 2001 [28]. The shrinkage occurring during photo-polymerization may cause secondary caries and repair failure [29]. Thus, developing compositions with minimal processing shrinkage is crucial for dentistry. Canellas et al. [30] showed incorporation of monofunctional MA-POSS into organic methacrylate-based dental matrixes resulted in decreased volumetric polymerization shrinkage, polymerization shrinkage stress, and wear. However, the resins’ flexural strength and modulus deteriorated upon the introduction of POSS. 

As mentioned before, substituents linked to a silica cage in POSS particles strongly influence its properties and the impact of POSS addition to polymer matrices. However, the functionality of POSS is yet another factor that will impact the properties of the obtained composites. While incorporating monofunctionalized POSS often leads to poor mechanical properties of the dental resins, exchanging it for an analogous three-functional one [31] or even fully functionalized (octa-armed) one [32] allows it to overcome this effect. POSS-crosslinked materials exhibit good mechanical properties due to silsesquioxane cage acting as reinforcement polymer network [33]. However, the authors stressed that too-high loading of POSS might result in deteriorated mechanical properties due to agglomeration of POSS [32].

Decreasing the shrinkage of dental resins may also be achieved by incorporating POSS functionalized with oxirane rings, such as epoxy-cyclohexyl POSS (E-POSS) [34]. Additionally, modification of dental resin with small amounts of E-POSS resulted in increased double bond conversion, decreased water uptake, and enhanced flexural strength.

Additional modification of MA-POSS allows silsesquioxane to act as a **co-initiator** during the polymerization of dental materials. The most commonly used co-initiator for cement resins mixed with photoinitiators is dimethylaminoethyl methacrylate (DMAEMA) [35]. Abbasi et al. [31] showed that modifying methacrylate POSS by anchoring tertiary amines on the methacrylate branches allows for obtaining aminated methacryl-POSS (AMA-POSS) as a novel co-initiator/crosslinker/reinforcing agent for dental resins. 

Although silsesquioxanes are mainly used as fillers or additives to dental resins, they can also act as **reactive binding primers** in restorative dentistry. For example, Raszewski and coworkers [36] used ethoxyhepta(isobutyl)octasilsesquioxane (*i*BU_7_-OEt POSS) to obtain fully substituted POSS (spherosilicates) functionalized with methacrylate and trimethoxysilyl groups. As shown by the authors, using spherosilicate-based coupling agents might allow for manufacturing restorative dental materials with improved bond strength and quality [36]. It is noteworthy that oligomeric silsesquioxanes, usually not used for dental materials, may constitute a group of **precursors** for the preparation of spherosilicates. A similar approach was taken by D. Wang et al. [37]—the authors used octavinyl-POSS as a precursor for the synthesis of acrylate-terminated octa-functionalized POSSPEGA macromonomer. The POSS-based macromonomer was used as a raw material for copolymerization with an acrylate-terminated triblock poly(ethylene glycol)-co-lactide (PEG-PLA) macromonomer to form POSS-PEG-PLA hydrogels for aiding alveolar bone repair adjacent to the periodontium. S. Burujeny et al. [38] used octakis-(3-glycidoxypropyl)-POSS (octaglycidyl-POSS) as a precursor for synthesizing triazolium-POSS. Triazolium-POSS, as an additive to dental resins, allowed not only for a higher conversion degree of methacrylate groups, but also led to composites that exhibited strong bactericidal properties and low hydrophilicity.

POSS particles were successfully used as **reinforcement and cell-proliferation-promoting agents** in scaffolds for bone regeneration, such as TMA-POSS [39], OEG_n_-POSS [40], octa-MA-POSS [41,42], and octamaleimic acid POSS [43]. It has been shown that POSS could also be used as additives to support the regeneration of skull bones [44] and facial bones, such as the mandible, maxilla, or alveolar bone. Chen et al. [41] showed that octamethacrylated silsesquioxanes could act as additives for biodegradable, bone-regenerative hydrogels. The authors showed that incorporating octa-MA-POSS enhanced the elastic modulus of studied hydrogels, allowed for higher water retention, and decreased the degradation rate. Later on, Chen and his group [45] presented scaffolds modified with octa-carboxyl POSS (OC-POSS) for repairing calvarial defects in rats where POSS acted as an agent **promoting cell proliferation** (3% POSS loading). Additionally, OC-POSS allowed for enhanced adhesion of vascular endothelial cells. POSS-based composites were also studied as promising materials for regenerating viscerocranium bones. Gong et al. [46] showed that incorporating octa-functionalized methacrylic POSS into scaffolds for alveolar bone regeneration allowed for obtaining material with enhanced mechanical properties due to additional crosslinking and the presence of a rigid silica-based cage. Moreover, composites exhibited higher hydrophilicity than the matrix, promoted cell adhesion, and cell regeneration, and **enhanced apatite-forming bioactivity**. In addition, composites modified with octa-MA-POSS were shown to reduce alveolar bone resorption induced by tooth extraction.

Types of POSS used in composites for dental applications are shown in Table 1. 

**Figure 3:**
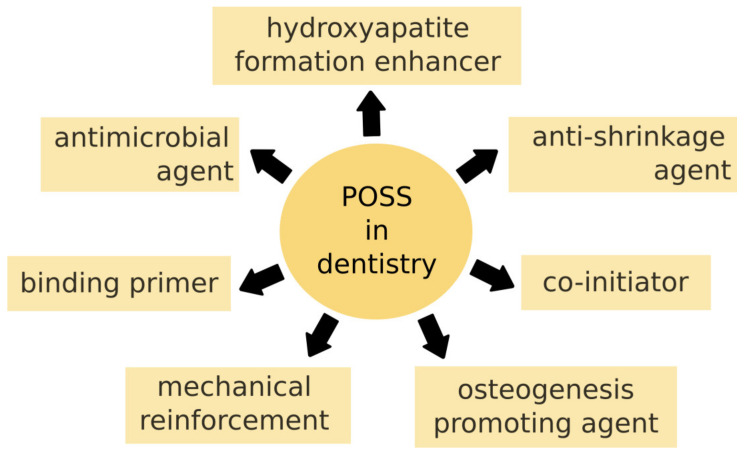
Application of POSS in dentistry.

Due to their versatility, POSS find various applications in dentistry (Figure 3). Moreover, the vast possibilities to modify already known POSS creates a wide range of new prospects. 

## 3. POSS Composites in Medicine and Dentistry

POSS may be modified by various substituents: reactive functional groups enable reactions with other active groups, while inert substituents ensure solubility and compatibility with the polymer matrix. Because of active/inactive substituents, various architectures may be obtained: (1) bead-like [48,49,50,51,52] when there are two reactive groups protruding directly from the opposing corners of the inorganic core, or when the POSS with an open cage structure is used, (2) pendant-like [50,53,54,55] when the two reactive groups are placed on one chain attached to the nanocage corner. Furthermore, POSS may modify previously obtained polymeric chains and might act as (1) side groups [56,57], (2) end groups [58,59] when POSS molecules are attached on both polymer ends, (3) crosslinkers [60,61] bonding two polymer backbones, or (4) net nodes [54,62,63] when POSS cage acts as a crosslinker. Finally, unreactive POSS may be physically blended into the polymeric matrix [64,65]. 

Despite various possible architectures available with use of POSS, in dental applications we noted that mainly octa- and mono-functionalized POSS are in use. Here, three main approaches are applied for manufacturing POSS-based composites. The first one is based on single-step polymerization of dental monomers with POSS; the second approach is obtaining POSS-containing macromonomers, followed by co-polymerization with dental monomers; the third approach is manufacturing POSS copolymers for in situ polymerization with dental monomers [66]. 

## 4. Dental Applications of POSS Composites

### 4.1. Endodontics

In endodontics, polymer composites are used as dental fillings, and they are mainly based on methacrylates. However, polymer fillings are usually combined with various fillers to improve their mechanical properties, such as fracture resistance, shrinkage during polymerization, and hardness. Moreover, hydrolysis resistance, and water absorption are the key parameters to ensure the long life of the products used, especially in a wet environment and in the presence of high compressive forces that are common in the oral cavity. Thus, the research is conducted to either obtain materials with better characteristics, or to find methods of repairing the fillings previously applied in the patient oral cavity. Self-healing and caries-preventive properties are achieved when material in use induces the growth of hydroxyapatite, which fills microcavities in the dental filling, or the material possesses bactericidal properties. Different kinds of POSS were found to be a promising solution to abovementioned problems [28,36,67].

#### 4.1.1. Marginal Integrity, Microleakage, and Shrinking Reduction

The most significant disadvantages of dental polymer composites are polymerization shrinkage and thermal expansion that is greater than the expansion of tooth tissues. The polymerization shrinkage of the composite material is responsible for forming internal stress in the material, leakage from between the filling and the cavity walls, and forming so-called postoperative hypersensitivity. Material shrinkage predominantly results in a leak at the gingival margin of a dentine or root cementum defect, since the adhesive forces to dentine and root cement are lower than those to the enamel. Regardless of the type of cavity and filling material, 80% to 90% of clinically diagnosed secondary caries are located in the gingival region of the tooth, and the finding of secondary caries is the most common reason for filling replacement [68]. Possible routes for shrinkage reduction may be divided into two categories: adjusting filler/matrix interaction (filler content, monomeric chemistry, monomeric structure, filler/matrix interactions, additives), and adjusting polymerization factors (polymerization rate, external constraint conditions, cavity geometry (C-factor), curing method, and placement technique) [69]. Hence, the important reason for introducing POSS as an additional component in a dental formulation is to address the problems of shrinkage and micro-leakage. 

The presence of a bulky POSS molecules limits the matrix’s free volume during the reaction, while the volume of the POSS nanocage does not change after the reaction. Consequently, the shrinkage of resins incorporated with POSS is decreased. Among functionalized POSS, MA-POSS has excellent compatibility with the methacrylate-based polymeric matrix. The shrinkage, double bond conversion, hardness, and mechanical resistance of the compositions based on with bisphenol A-glycidyl methacrylate (Bis-GMA), triethylene glycol dimethacrylate (TEGDMA), camphorquinone (CQ), and 2-(Dimethylamino)ethyl methacrylate (DMAEMA) with 0, 2, 5, and 10 wt.%. MA-POSS have been examined by Liu et al. [32]. The presence of 10 wt.% MA-POSS reduced shrinkage by about 27% (from 10.65% to 7.81%). Furthermore, the hardness and conversion of double bonds have increased (from 42.1% to 55% for 5 wt.% MA-POSS). On the contrary, mono-functional methacrylethyl-POSS (ME-POSS) has reduced flexural strength, while the shrinkage was only slightly reduced. The degree of double bond conversion was also reduced. The reason could be formation of POSS agglomerates as evidenced by SEM/EDS and AFM techniques. It is also the experience of our research team that multi-functionalized POSS tends to agglomerate less than monofunctional one, even if the full conversion of multi-functionalized POSS is more difficult to achieve [70]. Octa-functional MA-POSS with another inorganic filler (surface modified nano SiO_2_) was tested and the shrinkage was as low as 2.54 wt.% for 10 wt.% of MA-POSS, and 60 wt.%. of SiO_2_. Apparently, as for the shrinkage, high loading of POSS slightly over 10 wt.% is required. However, the highest hardness, Young modulus, and scratch resistance were obtained for 5 wt% POSS content.

MA-POSS may also be converted into amine-methacrylate POSS (AMA-POSS) through the aza-Michael reaction to obtain co-initiator-like POSS [31]. The influence of various amounts of converted methacrylate groups on shrinkage, degree of conversion, water sorption, and Young’s modulus was tested. The shrinkage was mainly influenced when all groups were substituted. However, the change was insignificant, and the other tested parameters showed that the best properties were achieved for silsesquioxane containing three converted groups. 

**Figure 4:**
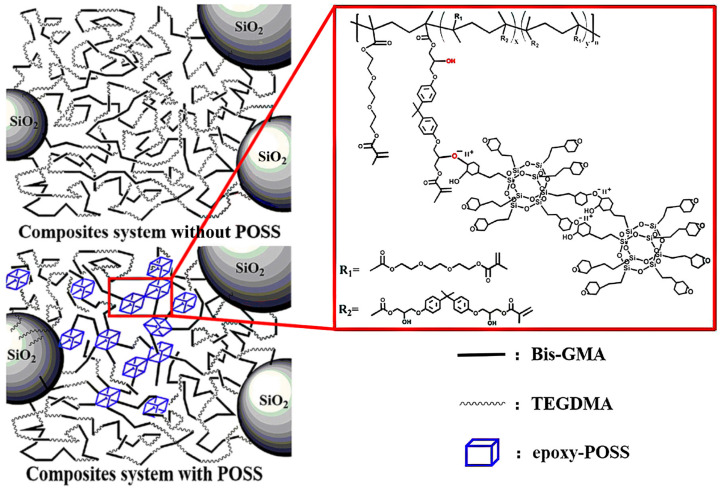
Schematic representation of dental composites system. Reprinted with permission from Li, Z.; Zhang, H.; Xiong, G.; Zhang, J.; Guo, R.; Li, L.; Zhou, H.; Chen, G.; Zhou, Z.; Li, Q. J. *Mech. Behav. Biomed. Mater.* **2020**, *103*, 103515 [34].

Besides MA-POSS and its modifications, AMA-POSS, the epoxycyclohexyl POSS (E-POSS), may also be used [34] (Figure 4). POSS might be bonded to the methacrylate matrix (Bis-GMA/TEGDMA) by the cationic ring-opening reaction. Its presence reduced the shrinkage to 2.91% for 5 wt. % of E-POSS (42% less compared to the reference material with no filler). A further increase in POSS loading had a lesser effect. Moreover, the water sorption has decreased, as well as the crosslink density, while the conversion of double bonds has increased. The lowest shrinkage (1.55%) was obtained for the composite containing only 2 wt.% E-POSS and 60 wt.% SiO_2_ filler. 

#### 4.1.2. Flexural Strength

Flexural strength is one of the essential properties of dental materials and a key value in product durability. Over the years, various fillers were used to increase flexural strength, and it was found that their amount, size, and distribution played a key role [71]. Among these fillers, nanosized additives with a hybrid organic–inorganic structure like POSS, show promising mechanical properties, as well as a positive contribution to esthetics [72]. 

**Figure 5:**
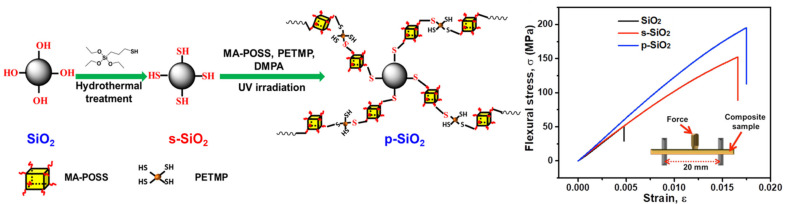
Schematic illustration of the fabrication of hybrid p-SiO_2_ particles and stress–strain curves of obtained composites. Reprinted with permission from Chen, H.; Wei, S.; Wang, R.; Zhu, M. *ACS Biomater. Sci. Eng.* **2021**, *7*, 1428–1437 [73].

By tailoring inert and reactive groups in POSS structure, it is possible to ensure good molecular solubility, and chemical bonding to the polymer matrix with exceptional distribution of nanosized molecules. Flexural strength was found to increase significantly for 10 wt.% of MA-POSS load, and to decrease at higher loadings, while the Young’s modulus was the highest for 2 wt.% of MA-POSS [28]. The reduction of flexural strength for higher POSS loadings originates from the lower conversion rate of the methacrylate double bonds. Lower conversion may result from the high functionality of MA-POSS, where some methacrylate groups could be inaccessible due to steric hindrances. Similar conclusions may be found in the works of Raszewski et al. [36], in which two kinds of POSS with four and two methacrylate substituents were used as a bonding material between an old, fractured composite and a new one. In both cases, the fracture induced in tests appeared in composites, not in the joint. POSS appear to be an excellent binder between methacrylates and filler surfaces. Chen et al. [73] synthesized materials with monodispersed SiO_2_ particles that were functionalized by MA-POSS, which greatly enhanced the flexural strength of obtained composite (Figure 5). Additionally, POSS may act as mechanical enhancement in other promising polymeric materials, such as non-isocyanate polyurethanes (NIPU). Incorporation of POSS resulted in an ~160% increase in tensile strength [74]. 

#### 4.1.3. Hydrolysis Resistance and Water Sorption

Water absorption is a key factor regarding hydrolytic stability. Methacrylate resins may be hydrolyzed, and thus their mechanical properties are reduced over time. The resistance towards hydrolysis is a factor determining long-term resilience. Moreover, water sorption creates an environment that promotes bacterial growth and may induce unfavorable effects such as swelling, plasticization, and oxidation [75]. 

Upon proper selection of substituents, it is possible to obtain POSS of low surface energy. Such POSS molecules are often used to increase the wetting angle of the composites or to obtain barrier properties towards gasses [76,77]. Therefore, they are candidates to reduce water absorption and extend product life by hydrolysis inhibition. Rizk et al. [78], in their study on POSS/bioactive glass adhesive materials, applied two kinds of POSS: 8-POSS with eight methacrylate groups, and 1-POSS with one methacrylate group, while the rest constituted of isobutyl substituents. Obviously, materials containing 1-POSS equipped with a large number of isobutyl substituents were more hydrophobic, and therefore the water sorption was reduced. However, in composites based on 8-POSS, those properties were slightly reduced due to the sorption inhibition by the higher crosslink density, or by POSS’ hydrophobic character (however, it was less hydrophobic than for 1-POSS). A similar effect was noticed by Canellas et al. [30], who used methacrylethyl-POSS (ME-POSS). 

#### 4.1.4. Antibacterial Materials

Considering the present, resin composite materials are the most commonly used materials for restoring hard tissue, i.e., enamel and dentin. Unfortunately, they are more prone to secondary caries, as the resin composites accumulate more dental plaque than other restorations and even the enamel [79]. Introducing antimicrobial properties allows for extending the life of resin composites. Polycationic antimicrobials are very efficient since they possess intense antimicrobial activity upon contact, which does not diminish or affect materials’ biocompatibility [80]. However, due to the addition of ionic compounds, water penetration between polymer chains increases, weakening the intermolecular interactions of the polymer network and causing plasticization, and as a result deterioration of mechanical properties is observed [81]. Deterioration of the mechanical strength of dental composites modified with ionic bactericidal compounds limits the broad application of this class of biomedical materials.

As mentioned above, POSS might be used as a hydrophobicity-inducing agent, which reduces the water sorption and degradation of the material in water conditions [30,37,82]. This approach guided Burujeny and co-workers, who modified standard polymer fillings with triazolium-POSS. The obtained nanocomposites were compared with quaternized dimethyl aminoethyl methacrylate monomer (DMAEMA-BC)-filled composites that are commonly used to obtain antimicrobial properties [38]. Such quaternized POSS, even with methacrylate units, have been present in literature for some time. However, they were not applied in dentistry [83,84,85]. Higher bactericidal activity of POSS-containing composites with simultaneous lower water uptake and higher flexural strength in wet conditions were observed. Nevertheless, the wet non-modified composite exhibited higher flexural strength, so there is still room for future advances. The exchange of some triazolium groups for more hydrophobic substituents would probably prove efficient, mainly if the POSS surface free energy decreased. Therefore, POSS would float more to the surface where they are mainly needed, becoming a hydrophobic layer with antimicrobial properties and not interfering with the polymeric chains. This modification may increase both the strength and bactericidal properties.

**Figure 6:**
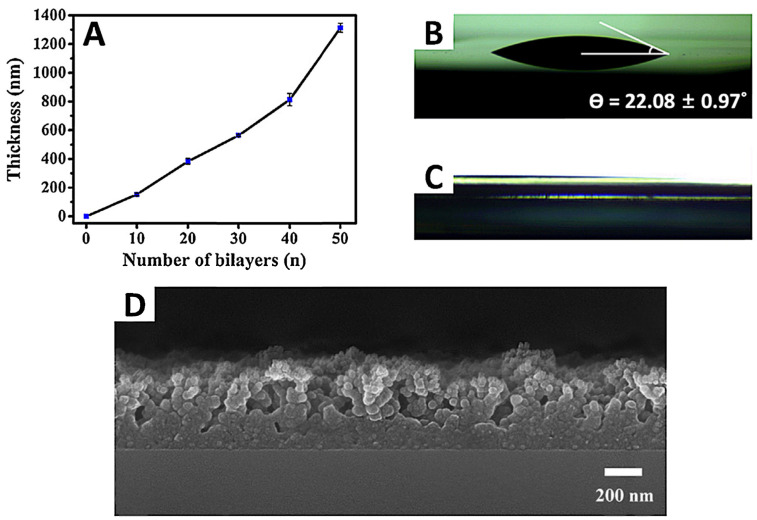
Characteristics of (BPEI/SiSQ)_n_ films. (**A**) Thickness growth curve of (BPEI/SiSQ)_n_ film (number of layers, *n* = 0, 10, 20, 30, 40, and 50). Static contact angle of (**B**) the bare silicon wafer as a control, (**C**) the (BPEI/SiSQ)_20_ film. (**D**) FE-SEM cross-sectional image of (BPEI/SiSQ)_20_ film. Reprinted with permission from Lin, X.; Hwangbo, S.; Jeong, H.; Cho, Y.A.; Ahn, H.W.; Hong, J. *J. Ind. Eng. Chem.* **2016**, *36*, 30–34 [86].

The antibacterial properties may also be obtained by formation of a superhydrophilic surface using silsesquioxane and branched polyethylenimine (BPEI) (Figure 6 [86]). The layer-by-layer surface exhibited enhanced tensile load, while its water angle contact was lower than 1° and did not increase the expression of the inflammatory cytokines. 

#### 4.1.5. Ca/P Ratio Stimulation

For better adhesion of dental fillings, adhesives are used, which form the so-called hybrid layer by diffusing and impregnating the subsurface of the pre-prepared dentin substrates. As mentioned earlier, dental composite materials (and the abovementioned layer) are susceptible to water sorption and hydrolytic/enzymatic degradation. It is possible to counteract this phenomenon by using calcium phosphate, hydroxyapatite, and bioactive glass nanoparticles to initiate the deposition of minerals on the hybrid layer and the remineralization of its surface. Unfortunately, such fillers increase the adhesive’s viscosity, causing limited wettability and promoting diffusion into the dentine. Additionally, unbound fillers are prone to leaching out and cause adverse effects [87].

POSS are known for their osteoinductive properties [39,41,46,54,70,88,89,90]. The silicon in the POSS core enables binding of calcium ions and triggers HAp deposition [44]. Additionally, they can be chemically bonded to the material by using appropriate substituents. Chemical bonding and the use of non-reactive substituents (consistent with the chemical nature of the matrix) enable straightforward homogenization of these materials. Therefore, POSS introduction might be a solution to hybrid layer demineralization. 

Rizk et al. [78] used two kinds of MA-POSS: octa- (POSS-8), and mono-functional (POSS-1) to examine the osteoinductive properties of POSS in dental composites through SEM/EDS spectroscopy after a 28-day incubation in artificial saliva. Moreover, a bioactive glass (BG-Bi) was tested, and the control was an unfilled sample. However, the BG-Bi particles were strongly aggregated in acetone up to micrometer sizes with various sizes, which were visible on DLS profiles. Unlike bioglass, both POSS types revealed good solubility in acetone dispersed in nanometric sizes POSS-1 and bioglass increased the viscosity. However, POSS-8 showed an increase in viscosity only at 20 wt.% loading. Most probably, the increase in viscosity was due to agglomeration. Moreover, the FTIR-measured conversion of double bonds was significantly reduced by POSS-1. Interestingly, all the fillers induced calcium phosphate deposition in the form of plate-like crystals. 

Further tests were carried out with different commercial adhesives [82], since all of them—except for one—contained inorganic fillers. Calcium phosphates deposited on them regardless of POSS-8 presence, except for the one that did not contain inorganic filler. POSS-8 presence in the later had successfully induced phosphate deposition in the adhesive without inorganic filler. Unfortunately, the calcium content was lower than that of HAp. The calcium ratio may increase when the material was in contact with simulated body fluid (SBF) [91]. All crystals had the appearance of cauliflower or plates—the structure of HAp present in enamel [91,92,93]. Recently, a similar study was conducted utilizing POSS-8 as a resin additive. POSS-8 induced osteoinductive properties, while the unmodified adhesive was not active [94].

### 4.2. Prosthodontics

The environment in which prosthetic is being placed, may give a rise to additional requirements for its properties. Direct contact with living organisms demands not only proper physicochemical properties but, above all, biological ones. Dentures are regarded as foreign bodies and may cause an undesired reaction from the organism. Thus, biocompatibility is one of most important requirements for such prostheses. POSS may find an application as a coating for various alloys used in prosthodontics or as a bone substituent and osteoblasts growth promoter.

#### 4.2.1. Corrosion Resistance

Corrosion of alloys in the oral cavity environment may be a pathogenic factor operating in various parts of the body. Therefore, dental alloys are subjected to high requirements: mechanical, physical, technological (which are essential from the point of view of their functional strength), and biological (necessary for their functioning in a living organism) ones. When two metals or metal alloys with different electrochemical potentials are present in the oral cavity, the dissolution of the metal alloy with lower potential may occur. Corrosion also occurs in the presence of one type of alloy due to the formation of the so-called local cell. It is a type of galvanic cell that takes place between different crystal systems of the alloy—the above is mainly observed for inhomogeneous (heterogeneous) alloys. The existence of galvanic currents in the oral cavity, related to the corrosion processes of the metals and their alloys, disturbs the physiological conditions of the functioning of living tissues and results in pathological changes called electrometalloses, which are one of the forms of prosthetic stomatopathies. Particularly undesirable are galvanochemical reactions of nickel-containing alloys, such as chromium–nickel alloy which is used for casting permanent dentures and for fusing porcelain. However, chromium–nickel alloys are prohibited in many countries, since a growing part of the population is allergic to nickel (from 16% in the USA to 22% in China [95,96]). Nickel allergy may manifest as stomatitis, lichen, gingivitis or periodontitis, burning tongue, tooth and jaw pain, and ailments when chewing food. It can also be more secretive and involve areas outside the mouth [97]. Therefore, if nickel alloys are to be used, they must be coated to protect against corrosion.

**Figure 7:**
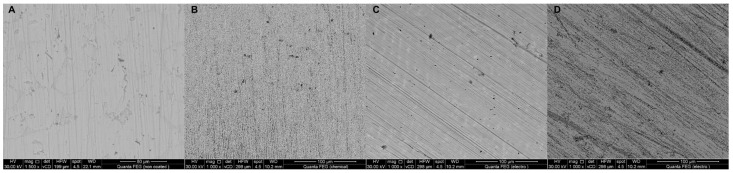
SEM images for (**A**) non-coated, (**B**) chemically coated, and (**C**) electrochemically coated Wirolloy with PVS, and (**D**) electrochemically nHAP coated alloy after immersion in artificial saliva for 14 days at 37 °C. Reprinted with permission from: Ghoneim, A.A.; Abdellatif, A.; Ameer, M.A. *Zeitschrift fur Anorg. und Allg. Chemie* **2019**, *645* [98].

Regardless of whether nickel is released or if there is a likelihood of galvanic currents, one should limit the contact between the metal alloy used and the saliva. The team that investigated this topic used standard dental alloy Ni-Cr (Wirolloy) and coated it with polyvinylsilsesquioxane (PVS) and nano-hydroxyapatite (nHAp) [98]. nHAp was deposited only electrochemically (with a current of ~1400 mV for two hours, and with a continuous CO2 stream to prevent the formation of carbonates on deposited film), while PVS was deposited electrochemically and through dip coating. The deposited vinyltrimethoxysilane (VTMS) was cured at 100 °C for 15 min to obtain the PVS layer. Then, dip coating was performed by immersing the alloy for the 30 s in pre-hydrolyzed VTMS solution. The electrochemical deposition was undergone in the same solution with a potential of −0.8 V for 200 s. SEM and EDS confirmed the even and homogeneous coating obtained using the electrochemical method. Finally, the obtained coated alloy behavior was examined in artificial saliva using Open Circuit Potential (OCP), Electrochemical Impedance Spectroscopy (EIS), and polarization techniques. PVS-dip-coated alloy exhibited superior corrosion resistance for the first three days of measurement; however, later it started to drop rapidly, and the film lost its protective properties. Conversely, electrochemically deposited layers showed high stability and corrosion protection, while SEM/EDS proved the formation of uniform surface of coating films on the electrode surface (Figure 7). The better adhesion of an obtained silsesquioxane layer could be achieved by the method that is used for the coating of stents [99]. Firstly, hydroxyl groups are introduced electrochemically on a metal surface, and after that a triethoxysilane functionalized with reactive groups bonding it to the next coat layer is applied. The over-layer may consist of octavinylsilsesquioxane or another kind of POSS that allows for a fine impenetrable layer. 

#### 4.2.2. Suppression of Alveolar Ridge Resorption

After tooth extraction, the alveolar walls undergo physiological resorption and remodeling, resulting in a variable dimension reduction. If the socket is not provided with augmentation material, the connective tissue undergoes increased proliferation, leading to a bone defect [100].

Additionally, alveolar trauma—which occurs as a result of tooth extraction, the inflammatory process, or a mechanical injury—results in the acceleration and increase of the bone resorption process [101]. Complete physiological regeneration of the post-extraction defect, consisting of filling with properly woven new bone, is rare. Excessive alveolar bone loss resulting from the performed extraction, exceeding the limits of physiological regeneration, may occur when vertical post-traumatic or post-inflammatory bone defects occur in the extraction area. 

Therefore, the techniques to preserve optimum dimensions and the alveolar ridge are needed. A powerful technique to prevent alveolar ridge resorption is to graft a bone substitute after tooth extraction. However, bone substitution materials are required to possess appropriate properties, such as mechanical strength, bioactivity, and osteoinductivity, simultaneously with tooth-like morphology. Many reports show non-toxicity of POSS [102] and POSS-containing materials [103,104,105,106,107,108,109] and drugs [49,110,111,112], or reduced cytotoxicity after POSS addition [54]. Moreover, POSS introduction is reported to induce hydroxyapatite (HAp) deposition, which results in osteoinductive properties [19,46,113,114,115,116]. Those osteoinductive properties may result from the self-organization of POSS-containing materials, resulting in porous morphology that facilitates cell proliferation [88,117].

Wang et al. [37] investigated POSS as a hydrophobicity modifier to reduce the degradation rate of hydrophilic, and thus degradation-prone, PEG/PLA (poly(ethylene glycol/polylactide) hydrogel designed to repair alveolar bone. Spontaneously emerged pores that varied between 0.4 µm and 4 µm were associated with POSS presence and its self-organization. The material was obtained starting from octavinyl-POSS that was converted into POSSCOOH by thiol-click reaction with 3-mercaptopro-pionic acid (MPA), its further esterification with PEG to produce POSSPEG, and finally via reaction with acryloyl chloride (ACC) to obtain reactive vinyl-ended star macromonomer POSSPEGA. The obtained macromonomer was then reacted with PEG, PLA/PEG copolymer, and diacrylate macromonomer to form an injectable, biodegradable hydrogel. Since it can be polymerized in situ, the surgical interference is minimal. Six materials with various POSS macromonomer concentrations were obtained, with POSS molar fraction ranging from 0 to 1. The presence of POSS resulted in improved swelling, reduced hydrolysis rate, and enlarged number of deposited fibroblasts.

**Figure 8:**
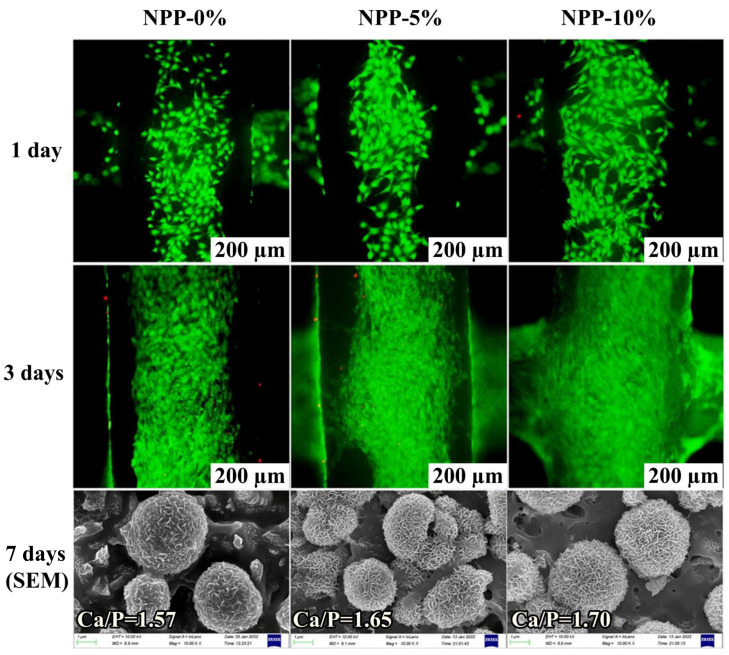
Cytocompatibility of different NPP scaffolds by co-culturing with MC3T3-E1 cells: live/dead staining; green fluorescence indicates viable cells and red fluorescence indicates dead cells. SEM images of the NPP-0%, NPP-5% and NPP-10% scaffolds after soaking in SBF for 7 days. Ca/P ratio obtained via EDS analysis. Reprinted with permission from Haihuan Gong; Yanyan Zhao; Qiwei Chen; Yilin Wang; Hong Zhao; Jing Zhong; Qing Lan; Ying Jiang; Wenhua Huang *J. Mater. Chem. B* **2022**, *10*, 8502–8513 [46].

Gong et al. [46] obtained a 3D-printed scaffold for alveolar ridge preservation. The filament for the 3D print consisted of nacre, polyurethane, and POSS (NPP). The polyurethane was obtained from isophorone diisocyanate (IPDI), polycaprolactone diol (PCL), and 2-hydroxyl methacrylate (HEMA). Therefore, it possessed reactive vinyl end-groups that were prone to UV-induced radical polymerization. The molar ratio PCL:IPDI:HEMA was kept constant at 1:2:2. The nacre was added as it contains signal molecules—bioactive agents that may facilitate bone growth—while MA-POSS was added in 0, 5, and 10 wt.% amounts. The incorporation of POSS in the system resulted in the reduction of the water contact angle. Since the used POSS possesses eight functional groups, it should increase the viscosity, yet the authors noticed decrease of viscosity in POSS-containing samples. Both properties are desirable in this application and for the planned 3D printing. Although they seem counterintuitive to how POSS behave in composites, it may occur when POSS have not yet undergone a radical reaction with methacrylates. POSS may influence the resin viscosity differently, depending on the type of functional groups, how POSS interact with the surrounding solvent, and the degree of POSS agglomeration [70,118]. Moreover, the presence of a small amount of POSS allowed for enhancement of composites’ compressive modulus. However, further loadings of POSS (up to 10 wt.%) resulted in reduced mechanical properties. The above may originate from formation of POSS agglomerates. Thus, POSS seem to be a good modifier of mechanical properties. This is crucial, as the mechanical properties should be tailored to the surrounding tissue to avoid mechanical mismatch [119,120,121]. Furthermore, the biocompatibility was also enhanced. The POSS-containing composites had accelerated growth of hydroxyapatite (HAp), as silicon in POSS may bind to Ca^2+^ ions and induce the deposition of HAp [44], which resulted in better MC3T3-E1 cells proliferation, and spindle-shaped morphology (Figure 8). Importantly, higher POSS loadings resulted in enhanced cell differentiation. Finally, the in vivo tests using rat models showed that the obtained scaffolds could effectively minimize the alveolar ridge resorption rate, as the labial width and height resorption rate were significantly reduced for materials incorporated with POSS.

### 4.3. Orthodontics

Orthodontics is an emerging field in which polymer composites are attracting growing attention. Thermoplastic materials are believed to have a great future utility in orthodontics, both as aligners and retainer appliances. In addition, orthodontists believe that smart materials with shape memory and self-cleaning or bactericidal properties are promising as materials for dentistry. Many of those properties were induced by incorporation of POSS into polymer matrix.

#### 4.3.1. Self-Cleaning Materials

One of the major concerns in orthodontics is plaque retention on brackets and microbial attachments onto biofilm [122]. One of the approaches to limit the plaque retention is to obtain the biocidal properties discussed before (in Section 4.1.4). Moreover, it is possible to prepare material that can clean itself of organic and inorganic precipitations. The “lotus effect” obtained with the microscopic bumps and superhydrophobic properties acquired this way is one of the most common ideas for self-cleaning materials. However, self-cleaning may also be induced by the presence of a superhydrophilic surface or through catalytic chemical reactions [123]. Microscopic bumps may be obtained by POSS’ self-organization on a material’s surface. 

In self-cleaning materials, POSS have been recognized to provide desired properties. Perfluoro/amino/isooctyl-POSS were found to act as a water and oil repellent in paints/lacquers [124]. The superhydrophobic surface was obtained by grafting poly(methyl silsesquioxane) PMSQ with long chains of ethyl 10-undecenoate [125], and finally, the tiny portions of OV-POSS were added to produce fabric with wetting angle as high as 168° [126]. The current state of art indicates that such composites merit further research. 

Self-healing materials are also demanded in orthodontics [123], as confirmed by a recent review article [127]. 

#### 4.3.2. Shape Memory Materials

Shape memory is the ability of a material to regain its original shape under the influence of a stimulus such as temperature, light, electric field, magnetic field, and chemical factors (i.e., pH, ionic strength, selective solvents, or chemical compounds). The mechanism of thermal shape memory in polymer composites results from thermal transitions of polymers, i.e., glass transition, characterized by the glass transition temperature *(T_g_*), or the melting process of the crystalline phase at a melting temperature (*T_m_*). Thermal shape memory in block copolymers is connected with existence of two microphases (two-blocks) of different thermal properties. The shape memory of POSS materials may be attributed to formation of the POSS microdomains via POSS mutual interactions that promote physically crosslinked networks in the material [51,128,129,130,131,132,133,134,135,136,137,138]. Materials with such properties can be used to produce transparent arch wires that have an initial minimal stiffness for easy handling, and for which the modulus of elasticity can change to a predetermined one upon exposure to light or body temperature [139]. However, there are none of these applications with the usage of POSS in dental applications described in the literature. It may be an interesting direction of research, especially as it is known in other fields of medicine [129,138,140,141]. It is worth mentioning that inducing a shape memory effect by POSS incorporation was reported previously for non-isocyanate polyurethanes (NIPU). Shape memory was obtained by chemical introduction of difunctional, double decker POSS, armed with cyclic carbonate groups, and thus obtaining the organic–inorganic poly(hydroxyl urethanes) (PHUs) with POSS bead-like cages stringed on the PHU chains [142]. Obtained composites exhibited improved thermomechanical properties and the ability to self-assemble into nanodomains in the size of 10–30 nm. The formation of the abovementioned microdomains allowed for physical crosslinking and shape memory properties. The strength loss due to programming the original shape at an elevated temperature is minimal (around ~10%).

### 4.4. Surgery

Facial bone surgery lies at the interface between surgery and dentistry. POSS osteoinductivity has received a lot of attention recently and has generated a large number of articles on tissue scaffolds and materials aiding bone healing. The wound healing aids, for example hydrogel dressings containing POSS, may also be interesting for dentistry. 

#### 4.4.1. Bone Reconstruction

POSS are well known for their complete non harm to biological systems, good biocompatibility, and osteoinductivity [46,55,70,88,102,132,143,144]. Therefore, it was only a matter of time until they found multiple applications in the field of bone regeneration. According to Scopus, the first article about POSS in the field of bone regeneration was written in the year 2013 [145]. It reported poly(vinyl acetate)/POSS (PVAc/POSS) electrospun nanofibers that were examined via scanning electron microscopy with energy-dispersive X-ray spectroscopy (SEM/EDS), X-ray photoelectron spectroscopy (XPS), transmission electron microscopy (TEM), electron probe microanalyzer (EPMA), X-ray powder diffraction (XRD), and thermogravimetric analysis (TGA). The analysis of deposited phosphates after SBF immersion and in vitro cytotoxicity on mouse myoblasts C2C1 was conducted. The unique porous morphology of the obtained electrospun mats was revealed, as well as their excellent biocompatibility and potential use as a substrate for proliferation and mineralization of osteoblasts. 

Since then, numerous articles about POSS in bone regeneration have been published [12,40,41,44,89,90,145,146]. Octa-TMA POSS were evenly dispersed with chitosan (CS) in water and acetic acid (1% *v*/*v*) [39]. Subsequently, the solutions were mixed, ultrasonicated, freeze-dried for 48 h, and finally neutralized and washed with distilled water. The obtained scaffolds were investigated for cytocompatibility, cell proliferation, alkaline phosphatase activity, osteocalcin production, and biomineralization assays. Results showed that the studied composites were cytocompatible with various cell lines, adhered and proliferated osteoblasts, and increased alkaline phosphatase (ALP) activity, osteocalcin secretion, and cell biomineralization. Another porous scaffold was obtained from poly-l-lactic acid (PLLA) doped with POSS-(PLLA)_8_ using thermally induced phase separation (TIPS) [147]. The bulk material, obtained by solvent casting and containing only 4 wt.% of POSS, was characterized by a six-fold increase of elongation at break, a 48% improvement of Young’s modulus, and a 56% increase of tensile strength, accompanied by excellent cell attachment, spreading, and proliferation [21]. In the next paper, the same material was cultured as a porous scaffold with miR-19b-3p-modified bone marrow mesenchymal stem cells (BMSCs) [147]. By transplanting, the scaffold was used for healing a critical-sized calvarial defect. After three months of recovery, immunohistochemical, pathology, and Micro-CT results showed that the BMSCs/PLLA/POSS scaffold had significantly facilitated the osteogenesis differentiation, enhanced the bone density of the defect area, and accelerated bone repair by suppressing the expression of ubiquitin enzyme, Smurf1. Suppressing of Smurf1is a new strategy for bone tissue engineering. Chitosan with POSS-(OH)_32_ was also studied in a gelatin methacryloyl (GelMA) matrix with in situ obtained calcium phosphate [44]. POSS served as a physical-chemical crosslinker to reinforce the hydrogel network and to improve mineralization capacity through bonding calcium ions to the silica cage. Therefore, mechanical properties, cell adhesion, and osteodifferentiation were enhanced. The same type of POSS (POH-POSS) was tested in an aqueous environment in the presence of human endothelial cells (HUVEC) [26]. Compared to the control group, the survival of cells incubated with 300 ppm POH-POSS was significantly increased (*p* < 0.05). Enlarged angiogenesis was observed in HUVEC treated with POH-POSS, which coincides with an intensification of the exosomal secretory pathway. POSS may also be used as macromonomers. The POSS core was surrounded by PEG substituents connected by disulfide bonds and two 2-ureido-4[1H]-pyrimidinone (UPy) groups and then crosslinked by thiol-click chemistry [90]. Compared to a non-filled sample, the improved mechanical properties of the obtained hydrogel and high biocompatibility, proliferation, adhesion, and osteogenesis of periodontal ligament stem cells (PDLSC) were noted. Importantly, a biological mechanism for improving PDLSC osteogenesis was also determined.

Combining chitosan and POSS-1 appears to be beneficial. Celesti et al. tested different concentrations of POSS-1 in CS-POSS hybrid hydrogels that were doped with ketoprofen to test drug release [148]. The positive effect of POSS-1 molecules on improving the drug release properties was revealed. Moreover, biological in vitro tests performed on human fetal osteoblastic cells (hFOB 1.19) demonstrated high biocompatibility, which was improved proportionally to POSS-1 concentration (0.5, 1, and 1.5 wt.%). Importantly, POSS-1 was attached by a covalent bond to chitosan by the Michael reaction. 

Similarly, covalent attachment of POSS were used in another work [146] in which POSS bearing one isoxazolidinyl group (isoxazolidinyl-POSS) had been grafted to chitosan by tert-butoxide-assisted amidation reaction; grafted chitosan was then crosslinked by genipin to obtain a hydrogel scaffold. Again, the drug release was determined, revealing a lower wt.% of ketoprofen released in time. Without POSS, the rate of the released drug was declining after 50 h. Both control and POSS-modified chitosan were biocompatible to hFOB1.19 cells; however, the hybrid material increased the percentage of dead cells by 12% on average.

**Figure 9:**
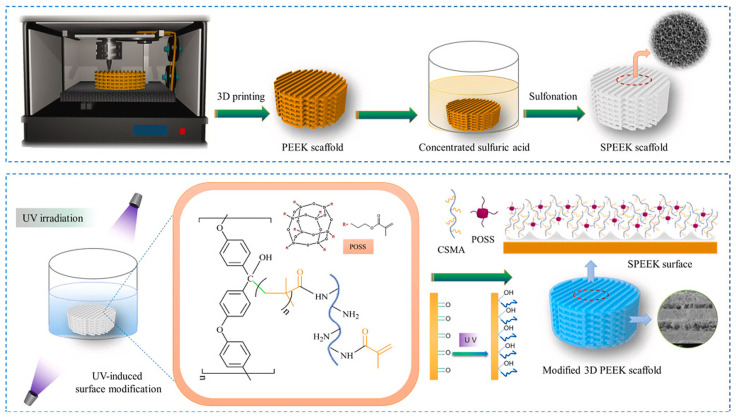
Schematic illustration of PEEK/POSS fabrication procedure. Reprinted with permission from: Liu, Z.; Zhang, M.; Wang, Z.; Wang, Y.; Dong, W.; Ma, W.; Zhao, S.; Sun, D. *Compos. Part B Eng.* **2022**, *230*, 109512 [47].

Finally, a macroporous polyetheretherketone (PEEK) scaffold modified with methacrylated chitosan and MA-POSS was obtained [47]. PEEK is a promising material considered a steel substitute in tooth implants; however, its interfacial bioactivity is poor. Macropores were obtained by PEEK sulfonation and 3D printing, while CS/MA-POSS presence contributed to micropores that promoted protein adsorption and apatite formation (Figure 9). In vitro and in vivo evaluation of the scaffold was performed using rat bone marrow mesenchymal stem cells (rBMSCs) and by further implantation into rat skulls. The results of the biological tests showed that the obtained structure and bioactive surface of the modified PEEK scaffolds provided suitable conditions for cell adhesion and proliferation, and promoted in vivo osteogenesis compared to scaffolds made of unmodified PEEK. 

#### 4.4.2. Wound Healing

In recent years, many publications have been devoted to hydrogels as materials for wound dressing. The above originates from hydrogel properties, such as adjustable mechanical properties that mimic natural tissue, good water absorption and retention capacity, controlled oxygen and nutrient permeability, easy and inexpensive processing, good wear resistance, and comfort. Modification of hydrogels with POSS allowed for obtaining bio-materials with superior properties. 

One of the first hydrogel systems containing POSS was obtained in the year 2005 [149] and then further developed [150,151]. Silver-containing hydrogels formed from diol-POSS, PEG, and LDI offer a prolonged antibacterial effect, preventing biofilm formation and infection. Materials with different properties were obtained. For example, dry cast films swell when immersed in water, while electrospun nanofiber mats shrink.

Such shrinking hydrogels were described by Bu et al. [152]. They are based on tetra-PEG modified with POSS and dopamine, as promising materials for closing wounds. High adhesive strength, a reduced swelling coefficient, and increased cell adhesion and growth were confirmed. Obtained hydrogels were tested in vivo on pigs. The incisions were quickly closed within 2 min, and the hydrogel layer remained stable after this time, proving to be less invasive and more comfortable than commonly used suturing methods. In addition, after 28 days, a significant acceleration of wound healing and complete degradation of the materials was demonstrated, which makes them even more attractive for clinical applications. The adhesion and shrinking properties played a key role here. It was reported that hydrogels that are crosslinked via hydrophobic crosslinkers, such as here with POSS, generally exhibit advantageous adhesive performance to tissues, especially those under wet conditions, such as lungs and blood vessels [153].

## 5. Conclusions and Future Outlooks

Polyhedral oligomeric silsesquioxanes are promising fillers for dental polymer composites. POSS incorporation not only may enhance resin properties, but also give rise to new ones, e.g., osteoinductivity. The latest works showed that POSS may be successfully incorporated into a polymer matrix to promote hydroxyapatite formation, reduce shrinkage during curing, increase the mechanical performance of the fillings, or ensure antimicrobial properties. Simultaneously, silsesquioxanes may act as co-initiators, which creates additional opportunities to combine the role of filler and a crosslinking agent. Selecting the proper POSS type and its content allows for tailoring the properties of dental composites.

Among all studied POSS for dental materials, POSS functionalized with methacrylate groups receive the most attention. Out of methacrylate silsesquioxanes, octamethacrylate POSS (MA-POSS) seems to be the most examined one. Its incorporation in dental resins reduces water sorption, enhances strength, and promotes osseointegration, reducing either the emerging cavities or microleakage, and may be used for bone reconstruction. Multiple reactive groups secure high homogeneity, and since the POSS nanocage is surrounded by long polymeric chains, the agglomeration effects are reduced. POSS concentration also plays an important role—most materials remained homogeneous or self-assembled into nanodomains when POSS concentration was less than 10 wt.%. Exceeding this content most often results in deterioration of mechanical properties such as flexural strength, hardness, Young’s modulus, and scratch resistance, which are a key properties in dental fillings. With these properties in mind, it would be good to use even 5 wt.% POSS. However, if controlling the shrinkage is the only issue to address, the amount of POSS may be increased up to 10 wt.%.

POSS hydrophobicity allows for the reduction of hydrolysis in both methacrylate dental fillings and PEG hydrogels composites. The above results in delaying their bio-degradation, e.g., until the alveolar bone is recovered. Thus, POSS may also act as a factor regulating the in vivo lifespan of dental materials. Moreover, hydrophobicity plays an important role in the case of tissue adhesives. Silsequioxanes might therefore be used as adhesives, since the strong adhesive effect on living tissue occurs due to the presence of the hydrophobic POSS segments. 

The future perspectives of POSS-containing materials in dentistry include new developments in the design of materials exhibiting shape memory, self-cleaning, and bactericidal properties. Another class of promising hybrid materials with POSS are injectable or 3D-printed scaffolds in which silsesquioxanes may ensure controlled microporous morphology and facilitate cell proliferation. Since the research focused mostly on octa-functional MA-POSS, many possible architectures that may be interesting due to various substituents (such as double-decker or open-cage POSS) have not been tested. One POSS molecule may play two or more different roles in a composite if equipped with different substituents. Combining POSS with chitosan, or HAp seems to be also attractive for self-repairing fillings, where osteoinductivity plays an important role. POSS self-assembling abilities may be used for drug release systems or enhancing the bactericidal properties of composites. Such approaches seem to be more advanced in other fields of medicine; therefore, taking advantage of these achievements and introducing them in dentistry might prove beneficial.

## Figures and Tables

**Table 1:** POSS in dental applications.

Type of POSS	Structure	Properties/Application	Ref.
Methacryl POSS (MA-POSS)	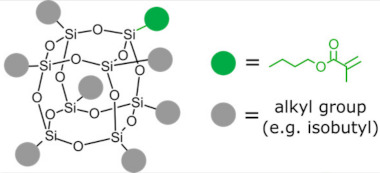	reduces the polymerization shrinkage in dental composites reduces water sorption and hydrolysis	[30,31,32,46,47]
	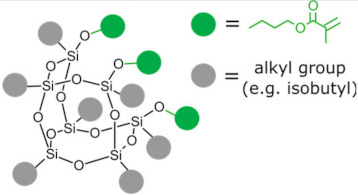	reduces the polymerization shrinkage in dental composites enhances mechanical properties in dental composites	
	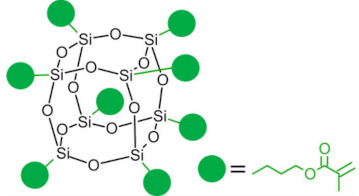	enhances mechanical properties of scaffold for bone regeneration enhances hydrophobicity promotes apatite-forming bioactivity in scaffolds	
Aminated methacryl-POSS (AMA-POSS)	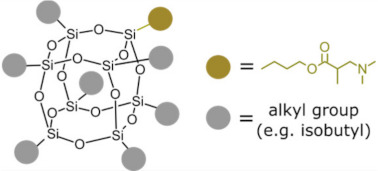	co-initiator, crosslinker, reinforcing agent	[31]
Epoxycyclohexyl POSS (E-POSS)	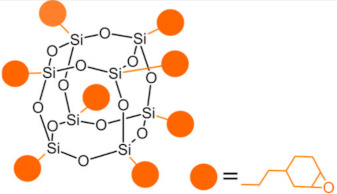	reduces shrinkage in dental composites decreases water uptake increases flexural strength	[34]
Octa-carboxyl POSS (OC-POSS)	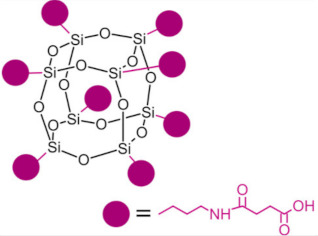	promotes cell proliferation in scaffolds for calvarial bone regeneration enhances adhesion of vascular endothelial cells	[45]
Octavinyl-POSS (OV-POSS)	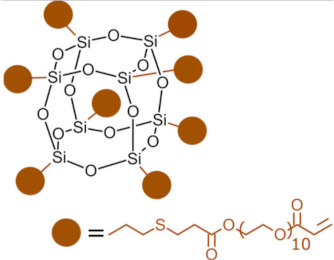	macromonomer in synthesis of hydrogels for aiding alveolar bone repair adjacent to the periodontium	[37]
Triazole-POSS	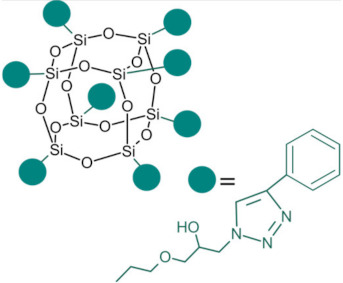	provides biocidal properties to the material, exhibits cytocompatibility reduces water sorption ensures higher flexural properties in wet conditions increases methacrylate conversion shows no effect on shrinkage	[38]
Fully functionalized POSS with methacrylate and trimethoxysilyl groups	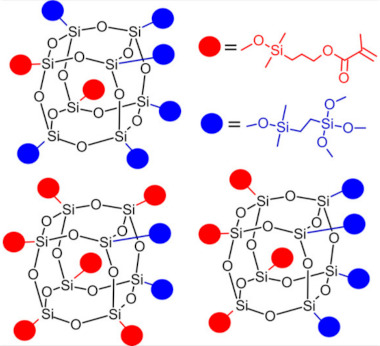	reactive binding primer	[36]

## Data Availability

Not applicable.

## References

[1] Fugolin A.P.P., Pfeifer C.S. (2017). New Resins for Dental Composites. J. Dent. Res..

[2] Samantaray R., Mohapatra A., Das S.S., Nanda K., Bharadwaj S. (2020). Polymers used in dentistry: An overview of literature. Indian J. Forensic Med. Toxicol..

[3] Robert P.M., Frank R.M. (1994). Periodontal Guided Tissue Regeneration With a New Resorbable Polylactic Acid Membrane. J. Periodontol..

[4] Katsarov P., Shindova M., Lukova P., Belcheva A., Delattre C., Pilicheva B. (2021). Polysaccharide-Based Micro- and Nanosized Drug Delivery Systems for Potential Application in the Pediatric Dentistry. Polymers.

[5] Prabha J.L., Roy A., Lakshmi T. (2018). Targeted drug delivery systems used in dentistry—A short review. Drug Invent. Today.

[6] Rokaya D., Srimaneepong V., Sapkota J., Qin J., Siraleartmukul K., Siriwongrungson V. (2018). Polymeric materials and films in dentistry: An overview. J. Adv. Res..

[7] Kadambi P., Luniya P., Dhatrak P. (2021). Current advancements in polymer/polymer matrix composites for dental implants: A systematic review. Mater. Today Proc..

[8] Hategekimana F., Kiraz N. (2022). Preparation and characterization of silica based nanoclusters as reinforcement for dental applications. Polym. Compos..

[9] Ha S.W., Weiss D., Weitzmann M.N., Beck G.R. (2019). Applications of silica-based nanomaterials in dental and skeletal biology. Nanobiomaterials in Clinical Dentistry.

[10] Wang C., Zhou L., Du Q., Shan T., Zheng K., He J., He H., Chen S., Wang X. (2022). Synthesis, properties and applications of well-designed hybrid polymers based on polyhedral oligomeric silsesquioxane. Polym. Int..

[11] Zheng Y., Gao Z., Han J. (2017). Current Chemistry of Cyclic Oligomeric Silsesquioxanes. Curr. Org. Chem..

[12] Liu S., Guo R., Li C., Lu C., Yang G., Wang F., Nie J., Ma C., Gao M. (2021). POSS hybrid hydrogels: A brief review of synthesis, properties and applications. Eur. Polym. J..

[13] Rozga-Wijas K., Sierant M. (2019). Daunorubicin-silsesquioxane conjugates (POSS-DAU) for theranostic drug delivery system: Characterization, biocompatibility and drug release study. React. Funct. Polym..

[14] Blanco I. (2021). Silicon-Containing Polymeric Materials. Polymers.

[15] Blanco I. (2018). Polyhedral oligomeric silsesquioxanes (POSS)s in medicine. J. Nanomed..

[16] Shi H., Yang J., You M., Li Z., He C. (2020). Polyhedral Oligomeric Silsesquioxanes (POSS)-Based Hybrid Soft Gels: Molecular Design, Material Advantages, and Emerging Applications. ACS Mater. Lett..

[17] Blanco I., Abate L., Bottino F.A. (2017). Mono substituted octaphenyl POSSs: The effects of substituents on thermal properties and solubility. Thermochim. Acta.

[18] Marcinkowska A., Prządka D., Andrzejewska E. (2019). POSS functionalized with mixed fluoroalkyl and methacryloxy substituents as modifiers for UV-curable coatings. J. Coat. Technol. Res..

[19] Fan L., Wang X., Wu D. (2021). Polyhedral Oligomeric Silsesquioxanes (POSS)-based Hybrid Materials: Molecular Design, Solution Self-Assembly and Biomedical Applications. Chin. J. Chem..

[20] Yuasa S., Sato Y., Imoto H., Naka K. (2018). Thermal Properties of Open-Cage Silsesquioxanes: The Effect of Substituents at the Corners and Opening Moieties. Bull. Chem. Soc. Jpn..

[21] Liu Z., Hu D., Huang L., Li W., Tian J., Lu L., Zhou C. (2018). Simultaneous improvement in toughness, strength and biocompatibility of poly(lactic acid) with polyhedral oligomeric silsesquioxane. Chem. Eng. J..

[22] Amna T., Hassan M.S., El-Newehy M.H., Alghamdi T., Abdulhameed M.M., Khil M.S. (2021). Biocompatibility Computation of Muscle Cells on Polyhedral Oligomeric Silsesquioxane-Grafted Polyurethane Nanomatrix. Nanomaterials.

[23] Jeong H.G., Han Y.S., Jung K.H., Kim Y.J. (2019). Poly(vinylidene fluoride) composite nanofibers containing polyhedral oligomeric silsesquioxane-epigallocatechin gallate conjugate for bone tissue regeneration. Nanomaterials.

[24] Loman-Cortes P., Huq T.B., Vivero-Escoto J.L. (2021). Use of Polyhedral Oligomeric Silsesquioxane (POSS) in Drug Delivery, Photodynamic Therapy and Bioimaging. Molecules.

[25] Huang L., Tan J., Li W., Zhou L., Liu Z., Luo B., Lu L., Zhou C. (2019). Functional polyhedral oligomeric silsesquioxane reinforced poly(lactic acid) nanocomposites for biomedical applications. J. Mech. Behav. Biomed. Mater..

[26] Feghhi M., Rezaie J., Akbari A., Jabbari N., Jafari H., Seidi F., Szafert S. (2021). Effect of multi-functional polyhydroxylated polyhedral oligomeric silsesquioxane (POSS) nanoparticles on the angiogenesis and exosome biogenesis in human umbilical vein endothelial cells (HUVECs). Mater. Des..

[27] Santulli C. (2019). Nanostructured Polymer Composites for Dental Fillings. Nanostructured Polymer Composites for Biomedical Applications.

[28] Fong H., Dickens S.H., Flaim G.M. (2005). Evaluation of dental restorative composites containing polyhedral oligomeric silsesquioxane methacrylate. Dent. Mater..

[29] Sonal, Kumar, S (2018). R.; Patnaik, A.; Meena, A.; Godara, M. Effect of adding nanosilica particulate filler on the wear behavior of dental composite. Polym. Compos..

[30] Canellas T.A.T., de Almeida Neves A., dos Santos I.K.B., de Rezende A.R.P., Fellows C.E., da Silva E.M. (2019). Characterization of low-shrinkage dental composites containing methacrylethyl-polyhedral oligomeric silsesquioxane (ME-POSS). J. Mech. Behav. Biomed. Mater..

[31] Abbasi M.R., Karimi M., Atai M. (2021). Modified POSS nano-structures as novel co-initiator-crosslinker: Synthesis and characterization. Dent. Mater..

[32] Liu Y., Wu X., Sun Y., Xie W. (2018). POSS dental nanocomposite resin: Synthesis, shrinkage, double bond conversion, hardness, and resistance properties. Polymers.

[33] Paszkiewicz S., Pawlikowska D., Szymczyk A., Dudziec B., Dutkiewicz M., Marciniec B., Linares A., Ezquerra T.A. (2018). Interfacial interactions in PTT–PTMO/polyhedral oligomeric silsesquioxane (POSS) nanocomposites and their impact on mechanical, thermal, and dielectric properties. Polym. Bull..

[34] Li Z., Zhang H., Xiong G., Zhang J., Guo R., Li L., Zhou H., Chen G., Zhou Z., Li Q. (2020). A low-shrinkage dental composite with epoxy-polyhedral oligomeric silsesquioxane. J. Mech. Behav. Biomed. Mater..

[35] Dos C., Lima R., Bandeira Da Silva D., Pino Vitti R., Miranda M.E., Cunha Brandt W. (2019). Mechanical properties of experimental resin cements containing different photoinitiators and co-initiators. Clin. Cosmet. Investig. Dent..

[36] Raszewski Z., Brząkalski D., Jałbrzykowski M., Pakuła D., Frydrych M., Przekop R.E. (2022). Novel Multifunctional Spherosilicate-Based Coupling Agents for Improved Bond Strength and Quality in Restorative Dentistry. Materials.

[37] Wang D.K., Varanasi S., Strounina E., Hill D.J.T., Symons A.L., Whittaker A.K., Rasoul F. (2014). Synthesis and characterization of a POSS-PEG macromonomer and POSS-PEG-PLA hydrogels for periodontal applications. Biomacromolecules.

[38] Burujeny S.B., Yeganeh H., Atai M., Gholami H., Sorayya M. (2017). Bactericidal dental nanocomposites containing 1,2,3-triazolium-functionalized POSS additive prepared through thiol-ene click polymerization. Dent. Mater..

[39] Tamburaci S., Tihminlioglu F. (2020). Chitosan-hybrid poss nanocomposites for bone regeneration: The effect of poss nanocage on surface, morphology, structure and in vitro bioactivity. Int. J. Biol. Macromol..

[40] Lu N., Lu Y., Liu S., Jin C., Fang S., Zhou X., Li Z. (2019). Tailor-Engineered POSS-Based Hybrid Gels for Bone Regeneration. Biomacromolecules.

[41] Chen M., Zhang Y., Xie Q., Zhang W., Pan X., Gu P., Zhou H., Gao Y., Walther A., Fan X. (2019). Long-Term Bone Regeneration Enabled by a Polyhedral Oligomeric Silsesquioxane (POSS)-Enhanced Biodegradable Hydrogel. ACS Biomater. Sci. Eng..

[42] Zhang Y., Chen M., Tian J., Gu P., Cao H., Fan X., Zhang W. (2019). In situ bone regeneration enabled by a biodegradable hybrid double-network hydrogel. Biomater. Sci..

[43] Ahmadipour S., Varshosaz J., Hashemibeni B., Safaeian L., Manshaei M., Sarmadi A. (2020). Calcitonin-loaded octamaleimic acid–silsesquioxane nanoparticles in hydrogel scaffold support osteoinductivity in bone regeneration. Pharm. Dev. Technol..

[44] Zhang X., He Y., Huang P., Jiang G., Zhang M., Yu F., Zhang W., Fu G., Wang Y., Li W. (2020). A novel mineralized high strength hydrogel for enhancing cell adhesion and promoting skull bone regeneration in situ. Compos. Part B Eng..

[45] Chen M., Zhang Y., Zhang W., Li J. (2020). Polyhedral Oligomeric Silsesquioxane-Incorporated Gelatin Hydrogel Promotes Angiogenesis during Vascularized Bone Regeneration. ACS Appl. Mater. Interfaces.

[46] Gong H., Zhao Y., Chen Q., Wang Y., Zhao H., Zhong J., Lan Q., Jiang Y., Huang W. (2022). 3D bio-printing of photocrosslinked anatomically tooth-shaped scaffolds for alveolar ridge preservation after tooth extraction. J. Mater. Chem. B.

[47] Liu Z., Zhang M., Wang Z., Wang Y., Dong W., Ma W., Zhao S., Sun D. (2022). 3D-printed porous PEEK scaffold combined with CSMA/POSS bioactive surface: A strategy for enhancing osseointegration of PEEK implants. Compos. Part B Eng..

[48] Song X., Zhang X., Li T., Li Z., Chi H. (2019). Mechanically Robust Hybrid POSS Thermoplastic Polyurethanes with Enhanced Surface Hydrophobicity. Polymers.

[49] Han Y., Xu C., Shi H., Yu F., Zhong Y., Liu Z., Loh X.J., Wu Y.L., Li Z., Li C. (2021). Engineered bio-adhesive polyhedral oligomeric silsesquioxane hybrid nanoformulation of amphotericin B for prolonged therapy of fungal keratitis. Chem. Eng. J..

[50] Wang M., Chi H., Joshy K.S., Wang F. (2019). Progress in the synthesis of bifunctionalized polyhedral oligomeric silsesquioxane. Polymers.

[51] Zhao B., Xu S., Adeel M., Zheng S. (2018). Formation of POSS-POSS interactions in polyurethanes: From synthesis, morphologies to shape memory properties of materials. Polymer.

[52] Xu S., Zhao B., Wei K., Zheng S. (2018). Organic–inorganic polyurethanes with double decker silsesquioxanes in the main chains: Morphologies, surface hydrophobicity, and shape memory properties. J. Polym. Sci. Part B Polym. Phys..

[53] Ozimek J., Sternik D., Radzik P., Hebda E., Pielichowski K. (2021). Thermal degradation of POSS-containing nanohybrid linear polyurethanes based on 1,6-hexamethylene diisocyanate. Thermochim. Acta.

[54] Hebda E., Bukowczan A., Michałowski S., Wroński S., Urbaniak P., Kaczmarek M., Hutnik E., Romaniuk A., Wolun-Cholewa M., Pielichowski K. (2020). Examining the influence of functionalized POSS on the structure and bioactivity of flexible polyurethane foams. Mater. Sci. Eng. C.

[55] Nezakati T., Tan A., Lim J., Cormia R.D., Teoh S.H., Seifalian A.M. (2019). Ultra-low percolation threshold POSS-PCL/graphene electrically conductive polymer: Neural tissue engineering nanocomposites for neurosurgery. Mater. Sci. Eng. C.

[56] Li K., Colonna S., Fina A., Monticelli O. (2019). Polyhedral oligomeric silsesquioxane (POSS) surface grafting: A novel method to enhance polylactide hydrolysis resistance. Nanomaterials.

[57] Morici E., Di Bartolo A., Arrigo R., Dintcheva N.T. (2018). POSS Grafting on Polyethylene and Maleic Anhydride-Grafted Polyethylene by One-Step Reactive Melt Mixing. Adv. Polym. Technol..

[58] Wu L., Magaz A., Maughan E., Oliver N., Darbyshire A., Loizidou M., Emberton M., Birchall M., Song W. (2019). Development data associated with effects of stiffness softening of 3D-TIPS elastomer nanohybrid scaffolds on tissue ingrowth, vascularization and inflammation in vivo. Data Br..

[59] Wu L., Magaz A., Maughan E., Oliver N., Darbyshire A., Loizidou M., Emberton M., Birchall M., Song W. (2019). Cellular responses to thermoresponsive stiffness memory elastomer nanohybrid scaffolds by 3D-TIPS. Acta Biomater..

[60] Xing C., Wang L., Xian L., Wang Y., Zhang L., Xi K., Zhang Q., Jia X. (2018). Enhanced Thermal Ageing Stability of Mechanophore in Polyurethane Network by Introducing Polyhedral Oligomeric Silsesquioxanes (POSS). Macromol. Chem. Phys..

[61] Wei B., Liu J., Ouyang L., Martin D.C. (2017). POSS-ProDOT crosslinking of PEDOT. J. Mater. Chem. B.

[62] Saha C., Behera P.K., Raut S.K., Singha N.K. (2020). Polyurethane–POSS hybrid materials: By solution blending and in-situ polymerization processes. Bull. Mater. Sci..

[63] Janeta M., Szafert S. (2017). Synthesis, characterization and thermal properties of T8 type amido-POSS with p-halophenyl end-group. J. Organomet. Chem..

[64] Szefer E., Stafin K., Leszczyńska A., Zając P., Hebda E., Raftopoulos K.N., Pielichowski K. (2019). Morphology, dynamics, and order development in a thermoplastic polyurethane with melt blended POSS. J. Polym. Sci. Part B Polym. Phys..

[65] Raftopoulos K.N., Hebda E., Grzybowska A., Klonos P.A., Kyritsis A., Pielichowski K. (2020). PEG-POSS Star Molecules Blended in Polyurethane with Flexible Hard Segments: Morphology and Dynamics. Molecules.

[66] Ghanbari H., Cousins B.G., Seifalian A.M. (2011). A nanocage for nanomedicine: Polyhedral oligomeric silsesquioxane (POSS). Macromol. Rapid Commun..

[67] Ghanbari H., Marashi S.M., Rafiei Y., Chaloupka K., Seifalian A.M., Hartmann-Thompson C. (2011). Biomedical Application of Polyhedral Oligomeric Silsesquioxane Nanoparticles. Applications of Polyhedral Oligomeric Silsesquioxanes.

[68] Mjor I.A. (1998). The location of clinically diagnosed secondary caries. Quintessence Int..

[69] Malhotra N., Kundabala M., Shashirashmi A. (2010). Strategies to overcome polymerization shrinkage--materials and techniques. A review. Dent. Update.

[70] Ozimek J. (2022). Elastomery Poliuretanowe, w Oparciu o 1,6-Heksametylenodiizocyjanian Modyfikowane POSS—Wpływ Architektury na Wybrane Właściwości Fizykochemiczne. Ph.D. Thesis.

[71] Gundogdu M., Kurklu D., Yanikoglu N., Kul E. (2015). The Evaluation of Flexural Strength of Composite Resin Materials with and without Fiber. J. Stem Cell Res. Ther..

[72] Ferracane J.L. (2011). Resin composite—State of the art. Dent. Mater..

[73] Chen H., Wei S., Wang R., Zhu M. (2021). Improving the Physical-Mechanical Property of Dental Composites by Grafting Methacrylate-Polyhedral Oligomeric Silsesquioxane onto a Filler Surface. ACS Biomater. Sci. Eng..

[74] MacInnis C.M., Younes G.R., Marić M. (2022). The effect of polyhedral oligomeric silsesquioxane fillers in non-isocyanate polyurethane hybrid resins. J. Appl. Polym. Sci..

[75] Kreutz M., Wiegand A., Stawarczyk B., Lümkemann N., Rizk M. (2021). Characterization of Methacrylate-Based Resins Containing Methacryl-Polyhedral Oligomeric Silsesquioxanes (MA-POSS-8). Materials.

[76] Gnanasekaran D., Ajit Walter P., Asha Parveen A., Reddy B.S.R. (2013). Polyhedral oligomeric silsesquioxane-based fluoroimide-containing poly(urethane-imide) hybrid membranes: Synthesis, characterization and gas-transport properties. Sep. Purif. Technol..

[77] Dou Q., Wang C., Cheng C., Han W., Thüne P.C., Ming W. (2006). PDMS-modified polyurethane films with low water contact angle hysteresis. Macromol. Chem. Phys..

[78] Rizk M., Hohlfeld L., Thanh L.T., Biehl R., Lühmann N., Mohn D., Wiegand A. (2017). Bioactivity and properties of a dental adhesive functionalized with polyhedral oligomeric silsesquioxanes (POSS) and bioactive glass. Dent. Mater..

[79] Pietrokovski Y., Nisimov I., Kesler-Shvero D., Zaltsman N., Beyth N. (2016). Antibacterial effect of composite resin foundation material incorporating quaternary ammonium polyethyleneimine nanoparticles. J. Prosthet. Dent..

[80] Melo M.A.S., Guedes S.F.F., Xu H.H.K., Rodrigues L.K.A. (2013). Nanotechnology-based restorative materials for dental caries management. Trends Biotechnol..

[81] Beyth N., Farah S., Domb A.J., Weiss E.I. (2014). Antibacterial dental resin composites. React. Funct. Polym..

[82] Rizk M., Pohle A., Dieckmann P., Tauböck T.T., Biehl R., Wiegand A. (2020). Mineral precipitation, polymerization properties and bonding performance of universal dental adhesives doped with polyhedral oligomeric silsesquioxanes. Int. J. Adhes. Adhes..

[83] Simionescu B., Ursu C., Cotofana C., Chibac A. Versatility of Silsesquioxane-Based Materials for Antimicrobial Coatings. Proceedings of the 1st International Electronic Conference on Materials.

[84] Simionescu B., Bordianu I.-E., Aflori M., Doroftei F., Mares M., Patras X., Nicolescu A., Olaru M. (2012). Hierarchically structured polymer blends based on silsesquioxane hybrid nanocomposites with quaternary ammonium units for antimicrobial coatings. Mater. Chem. Phys..

[85] Aflori M., Simionescu B., Bordianu I.-E.E., Sacarescu L., Varganici C.-D.D., Doroftei F., Nicolescu A., Olaru M. (2013). Silsesquioxane-based hybrid nanocomposites with methacrylate units containing titania and/or silver nanoparticles as antibacterial/antifungal coatings for monumental stones. Mater. Sci. Eng. B Solid-State Mater. Adv. Technol..

[86] Lin X., Hwangbo S., Jeong H., Cho Y.A., Ahn H.W., Hong J. (2016). Organosilicate based superhydrophilic nanofilm with enhanced durability for dentistry application. J. Ind. Eng. Chem..

[87] Tjäderhane L., Nascimento F.D., Breschi L., Mazzoni A., Tersariol I.L.S., Geraldeli S., Tezvergil-Mutluay A., Carrilho M.R., Carvalho R.M., Tay F.R. (2013). Optimizing dentin bond durability: Control of collagen degradation by matrix metalloproteinases and cysteine cathepsins. Dent. Mater..

[88] Ozimek J., Pielichowski K. (2022). Recent advances in polyurethane/poss hybrids for biomedical applications. Molecules.

[89] Du Y., Yu M., Chen X., Ma P.X., Lei B. (2016). Development of Biodegradable Poly(citrate)-Polyhedral Oligomeric Silsesquioxanes Hybrid Elastomers with High Mechanical Properties and Osteogenic Differentiation Activity. ACS Appl. Mater. Interfaces.

[90] Yu T., Zhang L., Dou X., Bai R., Wang H., Deng J., Zhang Y., Sun Q., Li Q., Wang X. (2022). Mechanically Robust Hydrogels Facilitating Bone Regeneration through Epigenetic Modulation. Adv. Sci..

[91] El-Damrawi G., Doweidar H., Kamal H. (2014). Characterization of New Categories of Bioactive Based Tellurite and Silicate Glasses. Silicon.

[92] Tauböck T.T., Zehnder M., Schweizer T., Stark W.J., Attin T., Mohn D. (2014). Functionalizing a dentin bonding resin to become bioactive. Dent. Mater..

[93] Zhou X., Sahai N., Qi L., Mankoci S., Zhao W. (2015). Biomimetic and nanostructured hybrid bioactive glass. Biomaterials.

[94] Paiva M.F., Rizk M., Pessan J.P., Kreutz M., Rohland B., Biehl R., Stadler A., Stellbrink J., Wiegand A. (2022). Material properties and bioactivity of a resin infiltrant functionalized with polyhedral oligomeric silsesquioxanes. Dent. Mater..

[95] Thyssen J.P., Linneberg A., Menné T., Johansen J.D. (2007). The epidemiology of contact allergy in the general population—Prevalence and main findings. Contact Dermat..

[96] Uter W., Hegewald J., Aberer W., Ayala F., Bircher A.J., Brasch J., Coenraads P.J., Schuttelaar M.L.A., Elsner P., Fartasch M. (2005). The European standard series in 9 European countries, 2002/2003—First results of the European Surveillance System on Contact Allergies. Contact Dermat..

[97] Wojciechowska M., Kołodziejczyk J., Gocki J., Bartuzi Z. (2008). Nickel hypersensitivity. Alerg. Astma Immunol..

[98] Ghoneim A.A., Abdellatif A., Ameer M.A. (2019). Electrochemical Behavior of Dental Ni-Cr Wirolloy Coated with Eco-friendly Films in Artificial Saliva. Z. Fur Anorg. Und Allg. Chemie.

[99] Bakhshi R. (2009). Coating Stent Materials with Polyhedral Oligomeric Silsesquioxane-Poly(Carbonate-Urea)urethane Nanocomposites. Ph.D. Thesis.

[100] Ashman A. (2000). Postextraction Ridge Preservation Using a Synthetic Alloplast. Implant Dent..

[101] Hansson S., Halldin A. (2012). Alveolar ridge resorption after tooth extraction: A consequence of a fundamental principle of bone physiology. J. Dent. Biomech..

[102] Siddiqui W.H., York R.G. (1993). Quaternary Silsesquioxane: A Developmental Toxicity Study in Rats. Toxicol. Sci..

[103] Misra R., Fu B.X., Morgan S.E. (2007). Surface Energetics, Dispersion, and Nanotribomechanical Behavior of POSS/PP Hybrid Nanocomposites RAHUL. J. Polym. Sci. Part B Polym. Phys..

[104] Adipurnama I., Yang M.C., Ciach T., Butruk-Raszeja B. (2017). Surface modification and endothelialization of polyurethane for vascular tissue engineering applications: A review. Biomater. Sci..

[105] Burke A., Hasirci N. (2004). Biomaterials: From Molecules to Engineered Tissues. Polyurethanes Biomed. Appl. New York Springer Sci. Bus. Media.

[106] Griffin M.F., Palgrave R.G., Seifalian A.M., Butler P.E., Kalaskar D.M. (2016). Enhancing tissue integration and angiogenesis of a novel nanocomposite polymer using plasma surface polymerisation, an in vitro and in vivo study. Biomater. Sci..

[107] Teirstein P.S. (2010). Editorial: Drug-eluting stent restenosis: An uncommon yet pervasive problem. Circulation.

[108] Kim S.K., Heo S.J., Koak J.Y., Lee J.H., Lee Y.M., Chung D.J., Lee J.I., Hong S.D. (2007). A biocompatibility study of a reinforced acrylic-based hybrid denture composite resin with polyhedraloligosilsesquioxane. J. Oral Rehabil..

[109] Punshon G., Vara D.S., Sales K.M., Kidane A.G., Salacinski H.J., Seifalian A.M. (2005). Interactions between endothelial cells and a poly(carbonate-silsesquioxane-bridge-urea)urethane. Biomaterials.

[110] Rizvi S.B., Yang S.Y., Green M., Keshtgar M., Seifalian A.M. (2015). Novel POSS-PCU Nanocomposite Material as a Biocompatible Coating for Quantum Dots. Bioconjug. Chem..

[111] Rizvi S.B., Yildirimer L., Ghaderi S., Ramesh B., Seifalian A.M., Keshtgar M. (2012). A novel POSS-coated quantum dot for biological application. Int. J. Nanomed..

[112] Iga A.M. (2008). Encapsulation of Novel Fluorescent Nanocrystals (Quantum Dots) with a Nanocomposite Polymer and Their Assessment by In-Vitro and In-Vivo Studies. Ph.D. Thesis.

[113] Zeinali R., Del Valle L.J., Torras J., Puiggalí J. (2021). Recent progress on biodegradable tissue engineering scaffolds prepared by thermally-induced phase separation (Tips). Int. J. Mol. Sci..

[114] Paradowska-Stolarz A., Wieckiewicz M., Owczarek A., Wezgowiec J. (2021). Natural Polymers for the Maintenance of Oral Health: Review of Recent Advances and Perspectives. Int. J. Mol. Sci..

[115] Ratner B.D. (2013). The Nature of Matter and Materials. Biomaterials Science: An Introduction to Materials.

[116] Jancia M. Synteza i Charakterystyka Elastomerów Poliuretanowych Modyfikowanych Poliedrycznymi Silseskwioksanami (POSS). Ph.D. Thesis.

[117] Kannan R.Y., Salacinski H.J., De Groot J., Clatworthy I., Bozec L., Horton M., Butler P.E., Seifalian A.M. (2006). The Antithrombogenic Potential of a Polyhedral Oligomeric Silsesquioxane (POSS) Nanocomposite. Biomacromolecules.

[118] Ozimek J. (2016). Effect of polyhedral oligomeric silsesquioxanes (POSS) on crystallization of polyoxytetramethylenediol Ocena wpływu rodzaju i ilości POSS na przebieg krystalizacji polioksytetrametylenodiolu. Przem. Chem..

[119] Birchall M.A., Herrmann P., Sibbons P. (2019). In vivo feasibility study of the use of porous polyhedral oligomeric silsesquioxane implants in partial laryngeal reconstruction. bioRxiv.

[120] Hortensius R.A., Ebens J.H., Harley B.A.C. (2016). Immunomodulatory effects of amniotic membrane matrix incorporated into collagen scaffolds. J. Biomed. Mater. Res. A.

[121] Weigert R. (2017). Implanted biomaterials: Dissecting fibrosis. Nat. Biomed. Eng..

[122] Jongsma M. (2015). Biofilm on Orthodontic Retention Wires: An In Vitro and In Vivo Study. Ph.D. Thesis.

[123] Hassan R., Umar M., Khan A., Abdullah A.M., Izwan S., Razak A., Duraccio D., Faga M.G., Gomez G., Ayala D. (2021). ’ A Review on Current Trends of Polymers in Orthodontics: BPA-Free and Smart Materials. Polymers.

[124] Jerman I., Koželj M., Orel B. (2010). The effect of polyhedral oligomeric silsesquioxane dispersant and low surface energy additives on spectrally selective paint coatings with self-cleaning properties. Sol. Energy Mater. Sol. Cells.

[125] Ahmed N., Zhang X., Fahad S., Jamil M.I., Aziz T., Husamelden E., Bittencourt C., Wan J., Fan H. (2020). Silsesquioxanes-Based Nanolubricant Additives with High Thermal Stability, Superhydrophobicity, and Self-cleaning Properties. Arab. J. Sci. Eng..

[126] Foorginezhad S., Zerafat M.M. (2019). Fabrication of superhydrophobic coatings with self-cleaning properties on cotton fabric based on Octa vinyl polyhedral oligomeric silsesquioxane/polydimethylsiloxane (OV-POSS/PDMS) nanocomposite. J. Colloid Interface Sci..

[127] Nowacka M., Kowalewska A. (2022). Self-Healing Silsesquioxane-Based Materials. Polymers.

[128] Yang X., Wang L., Wang W., Chen H., Yang G., Zhou S. (2014). Triple shape memory effect of star-shaped polyurethane. ACS Appl. Mater. Interfaces.

[129] Filion T.M., Xu J., Prasad M.L., Song J. (2011). In vivo tissue responses to thermal-responsive shape memory polymer nanocomposites. Biomaterials.

[130] Gu S., Xiefeng G. (2015). Method for Preparing Star-Shaped Biodegradable Shape-Memory-Polymer Nano Composite Material. China Patent.

[131] Bothe M., Mya K.Y., Jie Lin E.M., Yeo C.C., Lu X., He C., Pretsch T. (2012). Triple-shape properties of star-shaped POSS-polycaprolactone polyurethane networks. Soft Matter.

[132] Kazemi F., Mir Mohamad Sadeghi G., Kazemi H.R., Kazemi F., Kazemi H.R., Mir Mohamad Sadeghi G., Kazemi H.R. (2019). Synthesis and evaluation of the effect of structural parameters on recovery rate of shape memory polyurethane-POSS nanocomposites. Eur. Polym. J..

[133] Chatterjee T., Naskar K. (2018). Thermo-Sensitive Shape Memory Polymer Nanocomposite Based on Polyhedral Oligomeric Silsesquioxane (POSS) Filled Polyolefins. Polym. Technol. Mater..

[134] Bram A.I., Gouzman I., Bolker A., Eliaz N., Verker R. (2020). The Effect of POSS Type on the Shape Memory Properties of Epoxy-Based Nanocomposites. Molecules.

[135] Ishida K., Hortensius R., Luo X., Mather P.T. (2012). Soft bacterial polyester-based shape memory nanocomposites featuring reconfigurable nanostructure. J. Polym. Sci. Part B Polym. Phys..

[136] Jeon H.G., Mather P.T., Haddad T.S. (2000). Shape memory and nanostructure in poly(norbornyl-POSS) copolymers. Polym. Int..

[137] Alvarado-Tenorio B., Romo-Uribe A., Mather P.T. (2011). Microstructure and phase behavior of POSS/PCL shape memory nanocomposites. Macromolecules.

[138] Lee K.M., Knight P.T., Chung T., Mather P.T. (2008). Polycaprolactone-POSS chemical/physical double networks. Macromolecules.

[139] Eliades T. (2015). Orthodontic material applications over the past century: Evolution of research methods to address clinical queries. Am. J. Orthod. Dentofac. Orthop..

[140] Xu J., Song J. Biodegradable shape memory poly (ester-urethane) nanocomposites strengthened by polyhedral silsesquioxane (POSS) core. Proceedings of the 234th ACS National Meeting.

[141] Lin J. (2022). Shape Memory Polymer-Based Chitosan Nonwoven Fabric Material with Micro Pattern and Preparation Method Thereof 2019. Adv. Fiber Mater..

[142] Zhao B., Wei K., Wang L., Zheng S. (2020). Poly(hydroxyl urethane)s with Double Decker Silsesquioxanes in the Main Chains: Synthesis, Shape Recovery, and Reprocessing Properties. Macromolecules.

[143] Zaredar Z., Askari F., Shokrolahi P. (2018). Polyurethane synthesis for vascular application. Prog. Biomater..

[144] Crowley C., Klanrit P., Butler C.R., Varanou A., Platé M., Hynds R.E., Chambers R.C., Seifalian A.M., Birchall M.A., Janes S.M. (2016). Surface modification of a POSS-nanocomposite material to enhance cellular integration of a synthetic bioscaffold. Biomaterials.

[145] Ha Y.M., Amna T., Kim M.H., Kim H.C., Hassan S.S.M., Khil M.S. (2013). Novel silicificated PVAc/POSS composite nanofibrous mat via facile electrospinning technique: Potential scaffold for hard tissue engineering. Colloids Surfaces B Biointerfaces.

[146] Legnani L., Iannazzo D., Pistone A., Celesti C., GiofrèGiofrè S., Romeo R., Di Pietro A., Visalli G., Fresta M., Bottino P. (2020). Functionalized polyhedral oligosilsesquioxane (POSS) based composites for bone tissue engineering: Synthesis, computational and biological studies. RSC Adv..

[147] Xiong A., He Y., Gao L., Li G., Weng J., Kang B., Wang D., Zeng H. (2020). Smurf1-targeting miR-19b-3p-modified BMSCs combined PLLA composite scaffold to enhance osteogenic activity and treat critical-sized bone defects. Biomater. Sci..

[148] Celesti C., Iannazzo D., Espro C., Visco A., Legnani L., Veltri L., Visalli G., Di Pietro A., Bottino P., Chiacchio M.A. (2022). Chitosan/POSS Hybrid Hydrogels for Bone Tissue Engineering. Materials.

[149] Wu J., Ge Q., Burke K.A., Mather P.T. (2005). Crystallization of POSS in a PEG-based multiblock polyurethane: Toward a hybrid hydrogel. Mater. Res. Soc. Symp. Proc..

[150] Wu J., Hou S., Ren D., Mather P.T. (2009). Antimicrobial properties of nanostructured hydrogel webs containing silver. Biomacromolecules.

[151] Wu J., Ge Q., Mather P.T. (2010). PEG-POSS multiblock polyurethanes: Synthesis, characterization, and hydrogel formation. Macromolecules.

[152] Bu Y.Z., Sun G.F., Zhang L.C., Liu J.H., Yang F., Tang P.F., Wu D.C. (2017). POSS-modified PEG adhesives for wound closure. Chinese J. Polym. Sci..

[153] Du X., Liu Y., Wang X., Yan H., Wang L., Qu L., Kong D., Qiao M., Wang L. (2019). Injectable hydrogel composed of hydrophobically modified chitosan/oxidized-dextran for wound healing. Mater. Sci. Eng. C.

